# 4-Morpholine­carboxamidine

**DOI:** 10.1107/S1600536812042201

**Published:** 2012-10-13

**Authors:** Ioannis Tiritiris

**Affiliations:** a Fakultät Chemie/Organische Chemie, Hochschule Aalen, Beethovenstrasse 1, D-73430 Aalen, Germany

## Abstract

In the crystal structure of the title compound, C_5_H_11_N_3_O, the C=N and C—N bond lengths in the CN_3_ unit are 1.2971 (14), 1.3595 (14) (NH_2_) and 1.3902 (13) Å, indicating double- and single-bond character, respectively. The N—C—N angles are 115.49 (9)°, 119.68 (10)° and 124.83 (10)°, showing a deviation of the CN_3_ plane from an ideal trigonal–planar geometry. The morpholine ring is in a chair conformation. In the crystal, the mol­ecules are linked by N—H⋯N and N—H⋯O hydrogen bonds, generating a three-dimensional network.

## Related literature

For the synthesis of carboxamides by amidination of secondary amines with 4-benzyl-3,5-dimethyl-1*H*-pyrazole-1-carboxamidine hydro­chloride, see: Dräger *et al.* (2002 ▶). For the crystal structure of 4,4′-carbonyl-dimorpholine, see: Zhou *et al.* (2003 ▶).
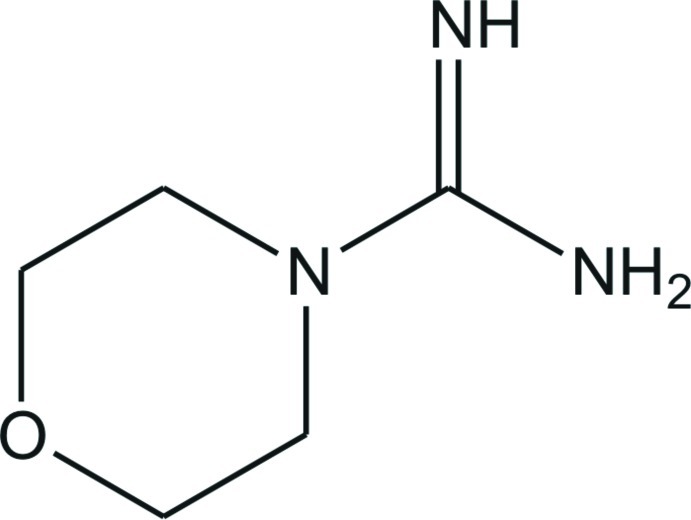


## Experimental

### 

#### Crystal data

C_5_H_11_N_3_O
*M* *_r_* = 129.17
Tetragonal, 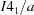

*a* = 16.5910 (6) Å
*c* = 9.7939 (3) Å
*V* = 2695.9 (2) Å^3^
*Z* = 16
Mo *K*α radiation
μ = 0.09 mm^−1^
*T* = 100 K
0.17 × 0.15 × 0.13 mm

#### Data collection

Bruker–Nonius KappaCCD diffractometer
2889 measured reflections
1628 independent reflections
1341 reflections with *I* > 2σ(*I*)
*R*_int_ = 0.022

#### Refinement

*R*[*F* ^2^ > 2σ(*F* ^2^)] = 0.037
*wR*(*F* ^2^) = 0.091
*S* = 1.06
1628 reflections
94 parameters
H atoms treated by a mixture of independent and constrained refinement
Δρ_max_ = 0.27 e Å^−3^
Δρ_min_ = −0.19 e Å^−3^

### 

Data collection: *COLLECT* (Hooft, 2004 ▶); cell refinement: *SCALEPACK* (Otwinowski & Minor, 1997 ▶); data reduction: *SCALEPACK*; program(s) used to solve structure: *SHELXS97* (Sheldrick, 2008 ▶); program(s) used to refine structure: *SHELXL97* (Sheldrick, 2008 ▶); molecular graphics: *DIAMOND* (Brandenburg & Putz, 2005 ▶); software used to prepare material for publication: *SHELXL97*.

## Supplementary Material

Crystal structure: contains datablock(s) I, global. DOI: 10.1107/S1600536812042201/zl2510sup1.cif

Structure factors: contains datablock(s) I. DOI: 10.1107/S1600536812042201/zl2510Isup2.hkl

Supplementary material file. DOI: 10.1107/S1600536812042201/zl2510Isup3.cml

Additional supplementary materials:  crystallographic information; 3D view; checkCIF report

## Figures and Tables

**Table 1:** Hydrogen-bond geometry (Å, °)

*D*—H⋯*A*	*D*—H	H⋯*A*	*D*⋯*A*	*D*—H⋯*A*
N2—H21⋯N1^i^	0.90 (2)	2.03 (2)	2.930 (1)	174 (1)
N2—H22⋯O1^ii^	0.88 (2)	2.13 (2)	3.007 (1)	174 (1)

Symmetry codes: (i) 
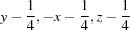
; (ii) 
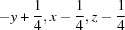
.

**Fig. 1.**
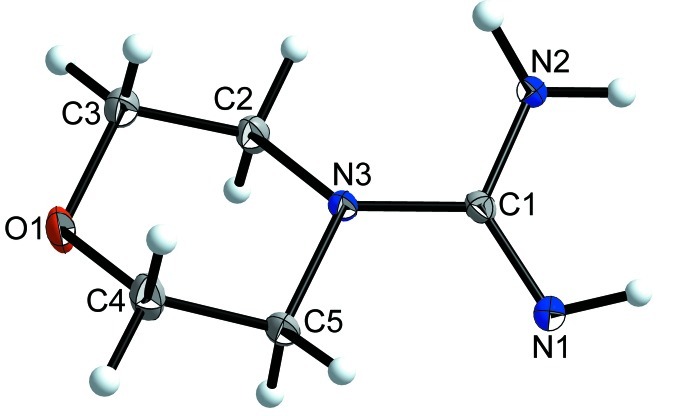
Molecular structure of the title compound with displacement ellipsoids at the 50% probability level.

**Fig. 2.**
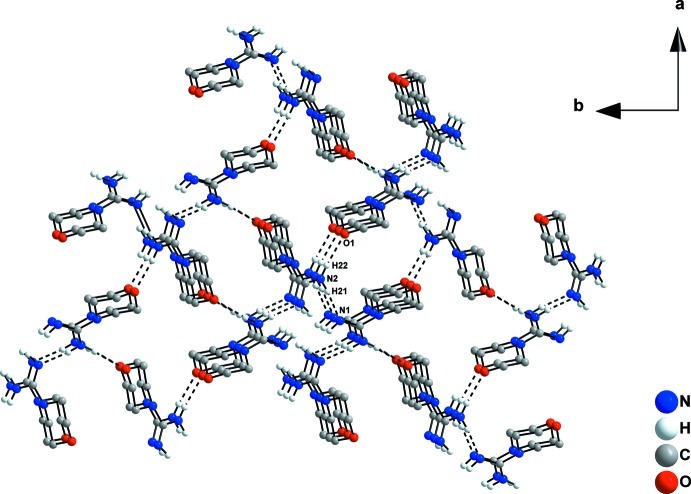
N–H···N and N–H···O hydrogen bonds between neighboring molecules, ab-view. The hydrogen bonds are indicated by dashed lines.

**Fig. 3.**
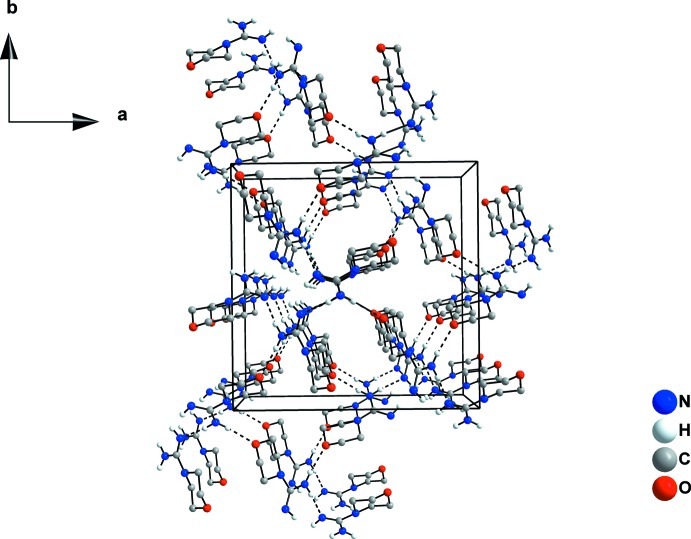
N–H···N and N–H···O hydrogen bonds generating a three-dimensional network, ab-view. The hydrogen bonds are indicated by dashed lines.

## supplementary crystallographic information

### Comment

4-Morpholine-carboxamidine is a guanidine derivative, bearing one morpholine moiety. It can be synthesized in a two-step synthesis by reacting first *O*-methylisourea sulfate with two equivalents of morpholine, giving the salt 4-morpholine-carboxamidinium sulfate (I). After deprotonation of (I) with a strong base, the carboxamidine can be isolated in good yield. Similar compounds have also been synthesized by amidination of secondary amines with 4-benzyl-3,5-dimethyl-1*H*-pyrazole-1-carboxamidine hydrochloride (Dräger *et al.*, 2002). In order to study the CO_2_ absorption ability of carboxamides, the title compound was prepared. Since its crystal structure was not known so far, it was decided to carry out an appropriate investigation. According to the structure analysis, the C1–N1 bond in the title compound is 1.2971 (14) Å, indicating double bond character. The bond lengths C1–N2 = 1.3595 (14) Å and C1–N3 = 1.3902 (13) Å are elongated and characteristic for a C–N amine single bond (Fig. 1). The N–C1–N angles are: 115.49 (9)° (N2–C1–N3), 119.68 (10)° (N1–C1–N3) and 124.83 (10)° (N1–C1–N2), showing a deviation of the CN_3_ plane from an ideal trigonal-planar geometry. The structural parameters of the morpholine ring in the here presented title compound agree very well with the data obtained from the X-ray analysis of the urea 4,4'-carbonyl-di-morpholine (Zhou *et al.*, 2003). In both crystal structures the morpholine rings adopt a chair conformation. Strong N—H···N and N—H···O hydrogen bonds between nitrogen atoms and oxygen atoms of neighboring molecules have been determined [*d*(H···N) = 2.03 (2) Å and *d*(H···O) = 2.13 (2) Å] (Tab. 1). Every carboxamidine molecule is sourrounded by four neighboring molecules (Fig. 2), generating a three-dimensional network (Fig. 3). In contrast, the imine hydrogen atom H11 is not involved in the hydrogen bonding system.

### Experimental

4-Morpholine-carboxamidinium sulfate (I) was prepared by heating one equivalent *O*-methylisourea sulfate with two equivalents of morpholine under reflux. The methanol formed in the reaction was distilled off and (I) precipitated in nearly quantitative yield. To a solution of 15.0 g (42 mmol) (I) in 50 ml water, a solution of 3.4 g (85 mmol) sodium hydroxide dissolved in 25 ml water was added dropwise under ice cooling. After warming to room temperature the aqueous phase was extracted with diethyl ether. The organic phase was finally dried over sodium sulfate. After evaporation of the solvent, the title compound precipitated in form of a colorless solid. Yield: 5.1 g (94%). On slow evaporation of an acetonitrile solution at room temperature, colorless single crystals suitable for X-ray analysis were obtained. ^1^H NMR (500 MHz, CD_3_CN/TMS): δ = 3.35–3.39 [m, 4 H, –CH_2_–], 3.74–3.78 [m, 4 H, –CH_2_–], 5.50 [s, 1 H, –NH], 6.35 [s, 2 H, –NH_2_]. ^13^C NMR (125 MHz, CD_3_CN/TMS): δ = 45.0 (–CH_2_–), 65.9 (–CH_2_–), 160.8 (C═N).

### Refinement

The N-bound H atoms were located in a difference Fourier map and were refined freely [N—H = 0.88 (2)–0.92 (2) Å]. The hydrogen atoms of the methylene groups were placed in calculated positions with d(C—H) = 0.99 Å. They were included in the refinement in the riding model approximation, with *U*(H) set to 1.2 *U*_eq_(C).

### Crystal data

**Table:**

C_5_H_11_N_3_O	*D*_x_ = 1.273 Mg m^−^^3^
*M**_r_* = 129.17	Melting point: 433 K
Tetragonal, *I*4_1_/*a*	Mo *K*α radiation, λ = 0.71073 Å
Hall symbol: -I 4ad	Cell parameters from 5017 reflections
*a* = 16.5910 (6) Å	θ = 0.4–28.3°
*c* = 9.7939 (3) Å	µ = 0.09 mm^−^^1^
*V* = 2695.9 (2) Å^3^	*T* = 100 K
*Z* = 16	Polyhedral, colorless
*F*(000) = 1120	0.17 × 0.15 × 0.13 mm

### Data collection

**Table:**

Bruker–Nonius KappaCCD diffractometer	1341 reflections with *I* > 2σ(*I*)
Radiation source: sealed tube	*R*_int_ = 0.022
Graphite monochromator	θ_max_ = 28.1°, θ_min_ = 2.5°
φ scans, and ω scans	*h* = −21→21
2889 measured reflections	*k* = −21→21
1628 independent reflections	*l* = −12→12

### Refinement

**Table:**

Refinement on *F*^2^	Primary atom site location: structure-invariant direct methods
Least-squares matrix: full	Secondary atom site location: difference Fourier map
*R*[*F*^2^ > 2σ(*F*^2^)] = 0.037	Hydrogen site location: difference Fourier map
*wR*(*F*^2^) = 0.091	H atoms treated by a mixture of independent and constrained refinement
*S* = 1.06	*w* = 1/[σ^2^(*F*_o_^2^) + (0.0306*P*)^2^ + 2.2674*P*] where *P* = (*F*_o_^2^ + 2*F*_c_^2^)/3
1628 reflections	(Δ/σ)_max_ < 0.001
94 parameters	Δρ_max_ = 0.27 e Å^−^^3^
0 restraints	Δρ_min_ = −0.19 e Å^−^^3^

### Special details

**Table:**

Geometry. All e.s.d.'s (except the e.s.d. in the dihedral angle between two l.s. planes) are estimated using the full covariance matrix. The cell e.s.d.'s are taken into account individually in the estimation of e.s.d.'s in distances, angles and torsion angles; correlations between e.s.d.'s in cell parameters are only used when they are defined by crystal symmetry. An approximate (isotropic) treatment of cell e.s.d.'s is used for estimating e.s.d.'s involving l.s. planes.
Refinement. Refinement of *F*^2^ against ALL reflections. The weighted *R*-factor *wR* and goodness of fit *S* are based on *F*^2^, conventional *R*-factors *R* are based on *F*, with *F* set to zero for negative *F*^2^. The threshold expression of *F*^2^ > σ(*F*^2^) is used only for calculating *R*-factors(gt) *etc*. and is not relevant to the choice of reflections for refinement. *R*-factors based on *F*^2^ are statistically about twice as large as those based on *F*, and *R*- factors based on ALL data will be even larger.

### Fractional atomic coordinates and isotropic or equivalent isotropic displacement parameters (Å^2^)

**Table:**

	*x*	*y*	*z*	*U*_iso_*/*U*_eq_	
N1	−0.14446 (6)	0.03950 (6)	0.30140 (11)	0.0159 (2)	
H11	−0.1813 (9)	0.0047 (9)	0.2645 (15)	0.019 (4)*	
N2	−0.06037 (6)	−0.03560 (6)	0.15536 (11)	0.0157 (2)	
H21	−0.1050 (9)	−0.0608 (9)	0.1248 (16)	0.022 (4)*	
H22	−0.0127 (10)	−0.0587 (9)	0.1491 (16)	0.025 (4)*	
N3	−0.00555 (5)	0.05719 (5)	0.30717 (10)	0.0125 (2)	
C1	−0.07396 (6)	0.02009 (6)	0.25455 (11)	0.0114 (2)	
C2	0.06247 (7)	0.07582 (7)	0.21681 (12)	0.0158 (3)	
H2A	0.0507	0.1252	0.1636	0.019*	
H2B	0.0711	0.0309	0.1519	0.019*	
C3	0.13724 (7)	0.08832 (7)	0.30179 (13)	0.0190 (3)	
H3A	0.1502	0.0379	0.3513	0.023*	
H3B	0.1832	0.1013	0.2413	0.023*	
O1	0.12587 (5)	0.15229 (5)	0.39777 (9)	0.0192 (2)	
C4	0.06078 (7)	0.13290 (8)	0.48713 (12)	0.0199 (3)	
H4A	0.0535	0.1772	0.5537	0.024*	
H4B	0.0742	0.0834	0.5387	0.024*	
C5	−0.01736 (7)	0.11994 (7)	0.40977 (12)	0.0170 (3)	
H5A	−0.0605	0.1036	0.4740	0.020*	
H5B	−0.0340	0.1708	0.3649	0.020*	

### Atomic displacement parameters (Å^2^)

**Table:**

	*U*^11^	*U*^22^	*U*^33^	*U*^12^	*U*^13^	*U*^23^
N1	0.0111 (4)	0.0149 (5)	0.0217 (5)	−0.0013 (4)	0.0005 (4)	−0.0051 (4)
N2	0.0106 (5)	0.0143 (5)	0.0222 (5)	0.0007 (4)	−0.0018 (4)	−0.0082 (4)
N3	0.0106 (4)	0.0134 (4)	0.0137 (5)	−0.0020 (3)	0.0023 (4)	−0.0056 (4)
C1	0.0129 (5)	0.0088 (5)	0.0126 (5)	−0.0004 (4)	−0.0008 (4)	−0.0002 (4)
C2	0.0142 (5)	0.0172 (5)	0.0160 (6)	−0.0041 (4)	0.0038 (4)	−0.0043 (4)
C3	0.0141 (5)	0.0197 (6)	0.0232 (6)	−0.0035 (4)	0.0029 (5)	−0.0050 (5)
O1	0.0168 (4)	0.0196 (4)	0.0213 (4)	−0.0088 (3)	0.0012 (3)	−0.0052 (3)
C4	0.0185 (6)	0.0260 (6)	0.0153 (6)	−0.0064 (5)	0.0011 (5)	−0.0064 (5)
C5	0.0145 (5)	0.0179 (6)	0.0185 (6)	−0.0014 (4)	0.0019 (4)	−0.0088 (5)

### Geometric parameters (Å, º)

**Table:**

N1—C1	1.2971 (14)	C2—H2B	0.9900
N1—H11	0.916 (15)	C3—O1	1.4302 (14)
N2—C1	1.3595 (14)	C3—H3A	0.9900
N2—H21	0.901 (16)	C3—H3B	0.9900
N2—H22	0.881 (16)	O1—C4	1.4268 (14)
N3—C1	1.3902 (13)	C4—C5	1.5169 (16)
N3—C5	1.4602 (14)	C4—H4A	0.9900
N3—C2	1.4671 (14)	C4—H4B	0.9900
C2—C3	1.5081 (16)	C5—H5A	0.9900
C2—H2A	0.9900	C5—H5B	0.9900
			
C1—N1—H11	107.8 (9)	C2—C3—H3A	109.5
C1—N2—H21	114.6 (10)	O1—C3—H3B	109.5
C1—N2—H22	119.6 (10)	C2—C3—H3B	109.5
H21—N2—H22	120.8 (14)	H3A—C3—H3B	108.1
C1—N3—C5	117.47 (9)	C4—O1—C3	109.61 (9)
C1—N3—C2	119.85 (9)	O1—C4—C5	111.87 (9)
C5—N3—C2	111.60 (9)	O1—C4—H4A	109.2
N1—C1—N2	124.83 (10)	C5—C4—H4A	109.2
N1—C1—N3	119.68 (10)	O1—C4—H4B	109.2
N2—C1—N3	115.49 (9)	C5—C4—H4B	109.2
N3—C2—C3	109.19 (9)	H4A—C4—H4B	107.9
N3—C2—H2A	109.8	N3—C5—C4	109.27 (9)
C3—C2—H2A	109.8	N3—C5—H5A	109.8
N3—C2—H2B	109.8	C4—C5—H5A	109.8
C3—C2—H2B	109.8	N3—C5—H5B	109.8
H2A—C2—H2B	108.3	C4—C5—H5B	109.8
O1—C3—C2	110.87 (9)	H5A—C5—H5B	108.3
O1—C3—H3A	109.5		
			
C5—N3—C1—N1	−2.87 (16)	N3—C2—C3—O1	−58.69 (12)
C2—N3—C1—N1	−143.83 (11)	C2—C3—O1—C4	60.42 (12)
C5—N3—C1—N2	177.42 (10)	C3—O1—C4—C5	−59.34 (13)
C2—N3—C1—N2	36.46 (14)	C1—N3—C5—C4	161.31 (10)
C1—N3—C2—C3	−160.75 (10)	C2—N3—C5—C4	−54.67 (12)
C5—N3—C2—C3	56.20 (12)	O1—C4—C5—N3	56.31 (13)

### Hydrogen-bond geometry (Å, º)

**Table:**

*D*—H···*A*	*D*—H	H···*A*	*D*···*A*	*D*—H···*A*
N2—H21···N1^i^	0.90 (2)	2.03 (2)	2.930 (1)	174 (1)
N2—H22···O1^ii^	0.88 (2)	2.13 (2)	3.007 (1)	174 (1)

Symmetry codes: (i) *y*−1/4, −*x*−1/4, *z*−1/4; (ii) −*y*+1/4, *x*−1/4, *z*−1/4.
